# Definition of acute kidney injury impacts prevalence and prognosis in ACS patients undergoing coronary angiography

**DOI:** 10.1186/s12872-021-01985-9

**Published:** 2021-04-15

**Authors:** Maren Weferling, Christoph Liebetrau, Daniel Kraus, Philipp Zierentz, Beatrice von Jeinsen, Oliver Dörr, Michael Weber, Holger Nef, Christian W. Hamm, Till Keller

**Affiliations:** 1 Department of Cardiology, Kerckhoff Heart and Thorax Center, Bad Nauheim, Germany; 2 German Centre for Cardiovascular Research (DZHK), Partner Site RheinMain, Bad Nauheim, Germany; 3 Medical Department I, Cardiology, University of Giessen, Giessen, Germany; 4 Medical Department I, Nephrology, University of Mainz, Mainz, Germany; 5 Department of Internal Medicine II, Hospital Darmstadt-Dieburg, Groß-Umstadt, Germany; 6 Department of Cardiology, Justus-Liebig-Universität Gießen, Campus Kerckhoff, Benekestr. 2-8, 61231 Bad Nauheim, Germany

## Abstract

**Background:**

Development of acute kidney injury (AKI) in invasively managed patients with acute coronary syndrome (ACS) is associated with a markedly increased mortality risk. Different definitions of AKI are in use, leading to varying prevalence and outcome measurements. The aim of the present study is to analyze an ACS population undergoing coronary angiography for differences in AKI prevalence and outcome using four established AKI definitions.

**Methods:**

944 patients (30% female) were enrolled in a prospective registry between 2003 and 2005 with 6-month all-cause mortality as outcome measure. Four established AKI definitions were used: an increase in serum creatinine (sCR) ≥ 1.5 fold, ≥ 0.3 mg/dl, and ≥ 0.5 mg/dl and a decrease in eGFR > 25% from baseline (AKIN 1, AKIN 2, CIN, and RIFLE definition groups, respectively).

**Results:**

AKI rates varied widely between the different groups. Using the CIN definition, AKI frequency was lowest (4.4%), whereas it was highest if the RIFLE definition was applied (13.2%). AKIN 2 displayed a twofold higher AKI prevalence compared with AKIN 1 (10.2% vs. 5.3% (*p* < 0.001)). AKI was a strong risk factor for mid-term mortality, with distinctive variability between the definitions. The lowest mortality risk was found in the RIFLE group (HR 6.0; 95% CI 3.7–10.0; *p* < 0.001), whereas CIN revealed the highest risk (HR 16.7; 95% CI 9.9–28.1; *p* < 0.001).

**Conclusion:**

Prevalence and outcome in ACS patients varied considerably depending on the AKI definition applied. To define patients with highest renal function-associated mortality risk, use of the CIN definition seems to have the highest prognostic relevance.

**Supplementary Information:**

The online version contains supplementary material available at 10.1186/s12872-021-01985-9.

## Introduction

Intravascular application of contrast media is believed to be one of the most frequent causes of hospital-acquired acute kidney injury (AKI) [1]. AKI after percutaneous coronary intervention (PCI), either in an elective or in an acute setting, is associated with higher rates of serious adverse cardiovascular events, dialysis, and death [2–4].

Comparison of prevalence and prognosis of patients developing AKI is challenging due to the different AKI definitions used in published studies, which are based on either relative or absolute changes in serum creatinine (sCR) or estimated glomerular filtration rate (eGFR) with varying thresholds [4]. To address this problem, an international consensus document with a standardized AKI definition was introduced in 2004 by the Acute Dialysis Quality Initiative (ADQI). Here AKI was defined as a decrease in eGFR of > 25% or as a minimum of a 1.5-fold increase in sCR (Risk, Injury, Failure, Loss, End-stage renal disease [RIFLE] classification) [5]. In 2007, the Acute Kidney Injury Network (AKIN) proposed a modification of the RIFLE criteria by defining AKI as a relative increase in sCR level of at least 1.5-fold or an absolute increase of at least 0.3 mg/dl [6]. This classification system was adopted by the international Kidney Disease: Improving Global Outcomes initiative (KDIGO) in 2012 [7] and is currently the most frequently used definition. A further definition of AKI was proposed in the context of contrast-induced nephropathy (CIN), introduced already in the 1980s, whereby AKI is defined as an absolute increase in sCr level of at least 0.5 mg/dl after contrast exposure or as a relative increase in sCR of > 25% [8]. Whereas most studies investigating AKI in the cardiological context utilize the AKIN or the RIFLE classification, in other studies the authors adhere to the CIN definition, indicating that there is still heterogeneity and uncertainty concerning the “right” AKI definition [1, 8–10]. Data on possible differences in the prevalence of AKI and, more importantly, in outcome according to these different definitions are scarce, especially in a cardiovascular study population. Thus, it is not known with certainty which AKI definition best reflects kidney-associated risk in ACS patients.

The aim of the present study was to evaluate AKI prevalence and outcome in a real-world ACS population using four different AKI definitions, with a specific focus on differences between patients with and without preexisting renal impairment. In addition, the association between contrast media (CM) use and AKI was examined in patients who underwent coronary angiography.

## Methods

### Study population

Between 2003 and 2005, n = 1023 patients were enrolled in the Bad Nauheim ACS registry at the Kerckhoff Heart and Thorax Center in Bad Nauheim, Germany. This registry has been described previously in more detail [11]. In brief, this all-comers registry included all patients who were referred for early coronary angiography or primary PCI due to ACS with an episode of chest pain within the preceding 48 h. The primary outcome measure was all-cause mortality within a median follow-up time of 200 days. For the present post-hoc analysis, 79 patients were excluded due to a lack of information on renal function on admission or before discharge or a lack of follow-up data, leading to a final cohort of 944 patients.

The study was conducted in adherence to the Declaration of Helsinki. All patients gave written informed consent and the study was approved by the local ethics board of the University of Giessen, Germany (AZ 145/11).

### Definition of renal function and AKI

Patients were stratified according to eGFR on admission into two groups based on the presence of chronic kidney disease (CKD): patients with an eGFR of at least 60 ml/min/1.73 m^2^ were defined as *non-CKD* patients and those with an eGFR below 60 ml/min/1.73 m^2^ were defined as *CKD* patients. This definition is based on the established KDIGO guidelines [7]. GFR was estimated by using the CKD-EPI formula. For the present analysis, four different definitions of AKI were evaluated:

1. *AKIN 1* definition: according to the AKIN definition with AKI defined as a relative increase in sCR of at least 1.5 fold (50% increase) from the baseline value;
2. *AKIN 2* definition: according to the AKIN definition with AKI defined as an absolute increase in sCR of at least 0.3 mg/dl;
3. *RIFLE* definition: according to the eGFR-based RIFLE classification with AKI defined as a decrease in eGFR of more than 25% from the baseline eGFR value;
4. *CIN* definition: according to the definition of AKI as an absolute increase in sCR of at least 0.5 mg/dl.

### Statistical analysis

Continuous variables are presented as mean with standard deviation (SD) or as median with interquartile range (IQR), as appropriate. Categorical variables are reported as absolute value and percentage. A two-sided chi-squared test was used in order to compare two groups (e.g. CKD and non-CKD) in terms of distribution of nominal variables. The Mann–Whitney U-Test was used to compare changes in eGFR and sCr between the CKD and non-CKD cohorts as well as before and after coronary angiography. Multivariate Cox regression was used to estimate hazard ratios (HR) for the different AKI definitions in terms of all-cause mid-term mortality, including adjustments for confounding factors such as CKD, age, and sex. For discrimination analysis, receiver operating curves (ROC) with area under the curve (AUC) were calculated for eGFR and AKI as well as CM volume and AKI for each definition. The strength of associations between AKI and CKD and AKI and high contrast volumes (≥ 300 ml) for each definition was assessed by calculating the odds ratios (OR) with 95% confidence interval (95% CI). Association between contrast volume and AKI was calculated by using Pearson’s correlation coefficient. All statistical tests were conducted with a level of significance (*p*-value) of < 0.05. SPSS Version 22.0 (IBM, Armonk, New York, USA) was used for all statistical analyses.

## Results

### Baseline characteristics

The study cohort comprised 944 patients (30% female). The mean (± SD) age was 64.1 ± 12.3 y (Table 1). On admission, 754 patients had preserved renal function (non-CKD), whereas 190 were classified as having CKD. Patients in the CKD group were generally older (72.4 ± 9.1 y) than the non-CKD group (62 ± 12.1 y; *p* < 0.001). There were more women in the CKD group (42.1% vs. 27.2%; *p* < 0.001).

**Table 1:** Baseline characteristics stratified according to the presence of chronic kidney disease on admission

	Entire cohort N (%)	non-CKD patients N (%)	CKD patients N (%)	p-value
N	944	754	190	
Female sex	285 (30.2)	205 (27.2)	80 (42.1)	< 0.001
Age, mean (SD), y	64.1 (12.3)	62 (12.1)	72.4 (9.1)	< 0.001
ACS				
STEMI	516 (54.7)	417 (55.3)	99 (52.1)	0.43
NSTEMI	237 (25.1)	196 (26.0)	41 (21.6)	0.21
UAP	191 (20.2)	141 (18.7)	50 (26.3)	0.02
Management				
Interventional	707 (74.9)	573 (76)	134 (70.5)	0.12
CABG	57 (6)	40 (5.3)	17 (9)	0.06
OMT	180 (19.1)	141 (18.7)	39 (20.5)	0.57
CVRF				
Art. HTN	631 (66.8)	481 (63.8)	150 (78.9)	< 0.001
Hyperlipidemia	401 (42.5)	318 (42.4)	83 (43.7)	0.71
Diabetes	208 (22)	144 (19.1)	64 (33.7)	< 0.001
Smoking status	300 (31.8)	277 (36.7)	23 (12.1)	< 0.001
Family history	139 (14.7)	119 (15.8)	20 (10.5)	0.07

CKD, chronic kidney disease; ACS, Acute Coronary Syndrome; STEMI, ST-segment elevation infarction; NSTEMI, non-ST-segment elevation infarction; UAP, unstable angina pectoris; CABG, coronary artery bypass graft; OMT, optimal medical therapy; CVRF, cardiovascular risk factors; Art. HTN, arterial hypertension

In the entire cohort 516 (54.7%) patients presented with ST-elevation myocardial infarction (STEMI), 237 (25.1%) with non-ST-elevation myocardial infarction (NSTEMI), and 191 (20.2%) had unstable angina pectoris (Table 1). STEMI and NSTEMI patients were equally distributed in the subgroups, with 55.3% vs. 52.1% (*p* = 0.43) and 26% vs. 21.6% (*p* = 0.21), respectively The CKD group contained more patients with unstable angina (18.7% vs. 26.3%; *p* = 0.02). There were no significant differences between CKD and non-CKD patients in the therapeutic approach to ACS taken after the initial diagnostic coronary angiography.

### Comparison of renal function on admission and at discharge

On admission, in the non-CKD cohort the mean sCr level was 0.84 ± 0.17 mg/dl, and the mean eGFR level was 89.41 ± 19.98 ml/min/1.73 m^2^ (Additional file 1: Table S1). For the CKD group these values were 1.53 ± 0.84 mg/dl and 45.47 ± 11.96 ml/min/1.73 m^2^, respectively (both *p* < 0.001). After coronary angiography, renal function in the non-CKD group measured before discharge worsened, showing a higher sCR level (0.93 ± 0.30 mg/dl) and a lower eGFR (81.86 ± 21.57 ml/min/1.73 m^2^) (both *p* < 0.001), whereas in the CKD group these parameters were nearly unchanged (1.54 ± 0.88 mg/dl, *p* = 0.02, for sCR and 48.43 ± 18.81 ml/min/m^2^, *p* = 0.93, for eGFR) (Additional file 1: Table S1).

### Development of AKI according to the different definitions

**Fig. 1:**
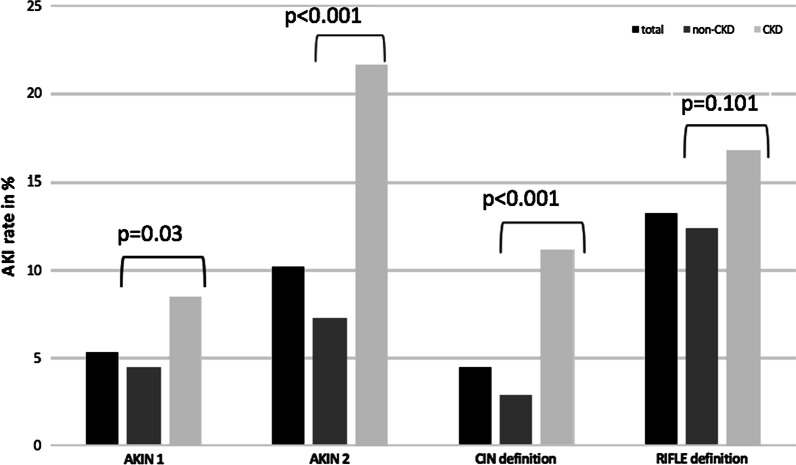
Incidence of AKI according to the four different definitions stratified by the presence of CKD. AKI rates are presented as percentages for the overall cohort (black column), non-CKD (dark-grey column) and CKD patients (light grey column) according to the four different AKI definitions, respectively. *P*-values are given for comparison of AKI rates of the CKD and non-CKD groups

Rates of AKI varied widely depending on the different AKI classification applied. According to the *AKIN 1* definition (sCr ≥ 1.5 times the baseline value), the AKI frequency was 5.3% overall, with 4.5% in the non-CKD group and 8.4% in the CKD group (*p* = 0.03) (Fig. 1; Additional file 1: Table S2). Application of the *AKIN 2* definition (sCR ≥ 0.3 mg/dl from baseline value), yielded almost two-fold higher AKI rates, with an overall rate of 10.2% (7.3% non-CKD vs. 21.6% CKD; *p* < 0.001). Using the *CIN* definition (sCr ≥ 0.5 mg/dl), the lowest overall AKI rate of 4.4% was detected (2.8% in the non-CKD group and 11.1% in the CKD group; *p* < 0.001). When applying the *RIFLE* definition (eGFR > 25%), the AKI rate overall was 13.2%, with similar rates between non-CKD and CKD patients (12.3% vs. 16.8%; *p* = 0.10). Hence, AKI occurred significantly more often in CKD patients than in non-CKD patients except when the *RIFLE* definition was applied. Figure 1 depicts the AKI rates of the different definitions for the entire cohort and the CKD and non-CKD groups.

The strongest association between CKD and AKI was observed for the *CIN* definition (OR 4.34; 95% CI 2.32–8.12), whereas the weakest association was found for the *RIFLE* definition (OR 1.44; 95% CI 0.93–2.23) (Table 2).

**Table 2:** Association of chronic kidney disease and development of acute kidney injury according to the four different AKI definitions

CKD versus non-CKD patients	Odds ratio	95% CI
AKI development according to		
AKIN 1 definition	1.95	1.05–3.61
AKIN 2 definition	3.50	2.25–5.44
CIN definition	4.34	2.32–8.12
RIFLE definition	1.44	0.93–2.23

CI, confidence interval; CKD, chronic kidney disease; AKI, acute kidney injury

Renal function at study entry, as determined by the continuous parameter eGFR, predicted the development of AKI with varying precision, depending on the AKI definition used. eGFR had the highest discriminatory ability when the *CIN* definition was used, with an AUC of 0.75 (95% CI 0.67–0.82; *p* < 0.001). In contrast, the eGFR had no predictive potential for AKI according to the *RIFLE* definition (AUC 0.5; 95% CI 0.44–0.56; *p* = 0.91) (Additional file 1: Table S3).

### AKI and prognosis

In the entire cohort, 62 (6.6%) patients died within the follow-up period (27 (3.6%) in the non-CKD group and 35 (18.4%) in the CKD group; *p* < 0.001). Overall, the mortality rate in patients who had developed AKI was higher in the CKD group than in the non-CKD group throughout all different AKI definitions: in non-CKD patients with AKI it ranged between 1.3 and 1.6% and in CKD patients between 3.7 and 11.1%, depending on the definition. Regarding mortality rates in relation to AKI status, according to *AKIN 1* 5% of patients without AKI died versus 34% of patients with AKI. Similarly, for *AKIN 2* these rates were 3.7% versus 32.3%, respectively. When the *CIN* definition was applied the highest death rates were observed: 4.4% for patients without AKI versus 52.4% for patients with AKI. The use of the *RIFLE* definition yielded mortality rates of 4.2% versus 8.2% for patients without and with AKI, respectively.

**Fig. 2:**
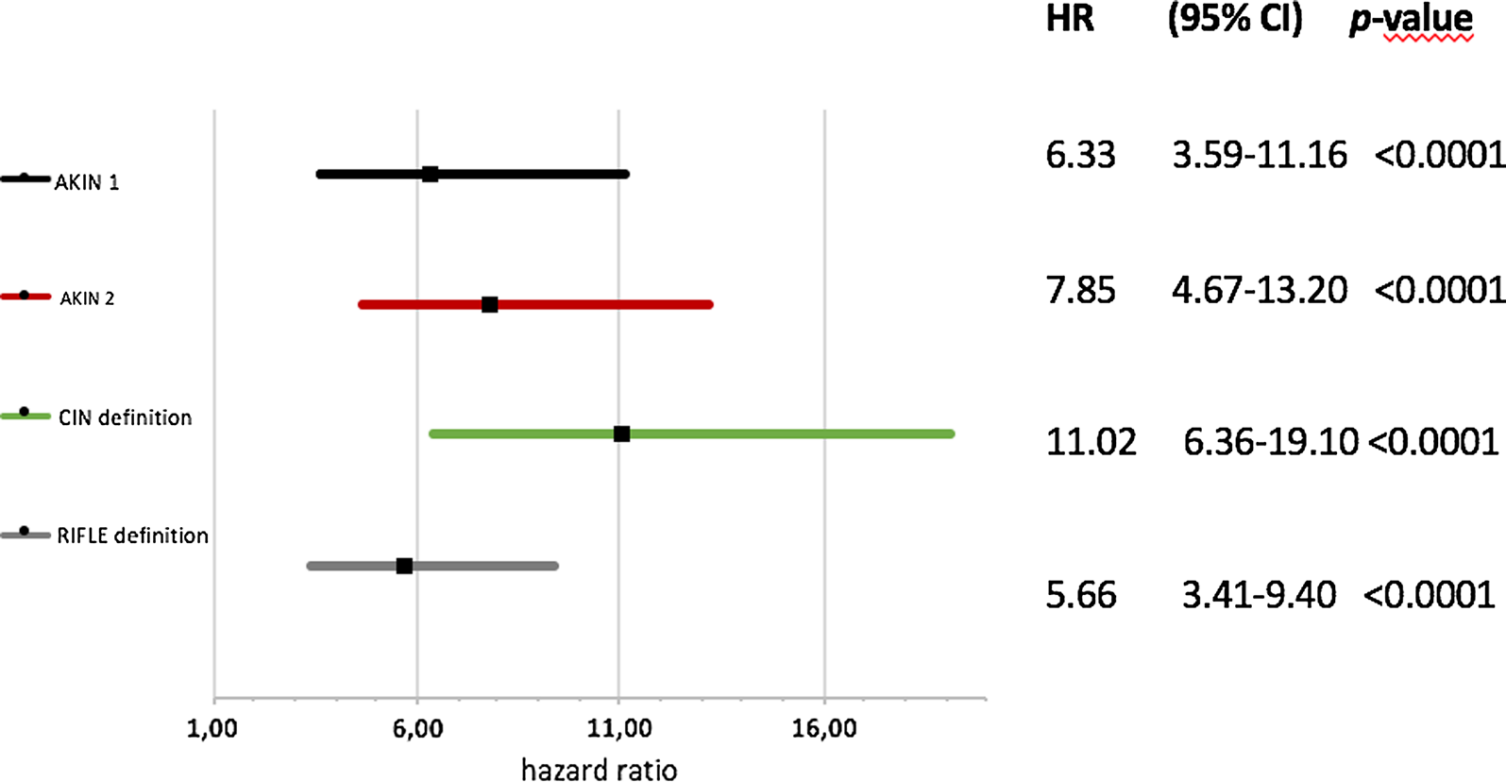
Development of AKI and mid-term mortaliy risk for the four different definitions. Association of AKI and 6-month mortality evaluated by hazard ratios with the corresponding 95% CI and level of significance (*p*-value) according to the four different AKI definitions after adjustment for CKD status, age and sex

Occurrence of AKI strongly predicted death in Cox regression analyses. This risk association differed between the AKI definitions. *CIN* showed the highest risk with a HR of 16.7 (95% CI 9.9–28.1; *p* < 0.001), which was 11.0 (95% CI 6.4–19.1; *p* < 0.001) after adjusting for CKD status, age, and sex (Fig. 2). Using *RIFLE* yielded the lowest HR of 6.0 (95% CI 3.7–10.0; *p* < 0.0001), which was 5.7 (95% CI 3.4–9.4; *p* < 0.0001) after adjustment.

### Association of contrast media volume and AKI

The median volume of CM used was 150 ml [IQR 100–200 ml] for the entire cohort. In the CKD group the median CM volume was not different than that used in the non-CKD group (150 ml [IQR 93–220] vs. 155 ml [IQR 110–200]; *p* = 0.60). Additional file 1: Figure S1 displays the distribution of CM volume used in patients with and without AKI according to the different definitions in CKD and non-CKD patients. No association between CM volume and development of AKI was found (Additional file 1: Table S4). Even the use of very high amounts of CM (≥ 300 ml) during coronary angiography showed no association with development of AKI, irrespective of the AKI definition applied (OR 0.65 [95% CI 0.23–1.9] for *AKIN 1*; OR 0.99 [95% CI 0.51–1.92] for *AKIN 2*; OR 0.80 [95% CI 0.28–2.28] for *CIN*; OR 0.79 [95% CI 0.42–1.49] for *RIFLE)*. Further, the amount of CM used, viewed as a continuous parameter, was not able to differentiate patients developing AKI from those patients without AKI, irrespective of AKI definition or CKD status (AUC values ranged between 0.48 and 0.52; see Table 3).

**Table 3:** Predictive value of the amount of contrast media to identify patients developing acute kidney injury stratified by CKD status

Contrast media volume	non-CKD patients			CKD patients		
	AUC	95% CI	*p*-value	AUC	95% CI	*p*-value
AKI development according to						
AKIN 1 definition	0.49	0.39–0.60	0.91	0.52	0.39–0.65	0.79
AKIN 2 definition	0.52	0.44–0.60	0.66	0.50	0.40–0.60	0.94
CIN definition	0.48	0.34–0.63	0.78	0.51	0.39–0.63	0.91
RIFLE definition	0.50	0.44–0.57	0.96	0.50	0.40–0.61	0.96

AUC, area under the curve; CI, confidence interval; CKD, chronic kidney disease; AKI, acute kidney injury

## Discussion

We analyzed four different established definitions of AKI with regards to prevalence of AKI and the prediction of mid-term risk in a real-world cohort of ACS patients undergoing coronary angiography with and without chronic renal impairment. Further, we evaluated the impact of the volume of CM on the development of AKI using the different AKI definitions. In summary, our results were as follows:

- First, AKI prevalence is clearly dependent on the definition used, with the highest prevalence observed when the *RIFLE* definition is applied.
- Second, the development of AKI was associated with an unfavorable outcome in ACS patients, and the strength of this association differed considerably according to the definition of AKI used, with the *CIN* definition being the strongest predictor of an unfavorable prognosis.
- Third, the volume of CM used during coronary angiography had no impact whatsoever on AKI rates, irrespective of baseline kidney function or AKI definition.

The most appropriate definition of AKI has been a matter of great debate over many years and is still a controversial issue. Due to the existence of different definitions of AKI with varying thresholds of renal parameters, prevalence and mortality rates as well as the strength of predictors of AKI showed a high variability even in similar disease entities, making a comparison difficult [5, 6, 12, 13]. The *AKIN* definition, which was adopted by the universal KDIGO guidelines, is currently the most frequently used AKI definition, especially in the context of cardiovascular patient care and cardiovascular research [2, 14]. Nevertheless, older definitions of AKI, such as those relating to CM exposure like the *CIN* definition, are still in use. The KDIGO working group strongly recommends using the KDIGO-based AKI definition for contrast media-induced acute kidney injury (CI-AKI) and for all other AKI forms [15]. This recommendation is also in line with the guidelines of Contrast Media Safety Committee (CMSC) and of the European Renal Best Practice (ERBP) working group [12, 16]. With this information as a backdrop, our findings favoring the *CIN* definition as the strongest risk predictor over the *AKIN* definitions (KDIGO) are highly relevant.

According to the *AKIN* definition of AKI, absolute as well as relative changes in sCR can be used optionally as an “either-or” criterion. However, results of studies are highly variable with regard to the prognostic value and the prevalence of AKI, depending on either absolute or relative changes in sCR [16–18]. We observed differences in AKI prevalence between the relative and the absolute *AKIN* definitions, with AKI rates almost twice as high for the *AKIN 2* (absolute) compared with the *AKIN 1* (relative) definition (10.2% vs. 5.3%). Mid-term outcome was unfavorable for both definitions, which agrees with the findings of other studies using those definitions [19–21]. To the best of our knowledge, a direct comparison within the *AKIN* definition between an absolute change in sCR levels versus a relative change has not been published to date.

The low prevalence of AKI of 4.4% observed in our cohort when using the *CIN* definition is comparable to AKI rates for that particular definition found in other studies within the context of coronary angiography (the majority being ACS patients) [22, 23]. As with *AKIN*, AKI can be defined with the *CIN* definition either by an absolute change in sCR, which we used for our analysis, or by a relative change of at least 25% from the baseline value [8]. Studies that used both the relative and the absolute (“either-or”) definition of *CIN* observed considerably higher AKI rates ranging between 12.7 and 18.3%, raising concerns that AKI prevalence might then be overestimated [10, 19].

Of the four AKI definitions evaluated, the *CIN* definition best identified patients at highest risk regarding 6-month mortality. This result appears to contradict the findings described by Silvain et al. [19]. Here, in an ACS cohort comparing different AKI definitions, the *CIN* definition showed the lowest predictive ability with respect to 1-year mortality compared with the *AKIN* and *RIFLE* definitions. The fact that the authors used the “either-or” *CIN* definition may have weakened the prognostic value of this definition. Budano et al. compared both *CIN* definitions in a PCI cohort in terms of adverse in- and out-of-hospital events and found the absolute rise in sCR to be more predictive than the relative increase, despite the fact that AKI rates in the latter were more than twice as high (6.9% vs.15.9%) [17]. These findings suggest that the more “liberal” *CIN* definition using relative changes in sCR tends to overestimate AKI rates and consequently underestimates mortality risk.

The eGFR-based *RIFLE* definition has been questioned in the past due to the lack of an equilibrium of sCR in AKI as a mandatory condition for an sCR-based estimation of the GFR [4]. Our findings support the application of sCR-based over eGFR-based criteria to define AKI, as the eGFR-based *RIFLE* definition showed the highest AKI rates but the lowest association with 6-month all-cause mortality. Thus, use of the *RIFLE* definition to define AKI in invasively managed ACS patients might misclassify patients as being more ill than they actually are.

A further focus of our study was the evaluation of CM volume and its impact on AKI rates. CM is known to have nephrotoxic effects due to parenchymal ischemia, tubular cell injury, and damage to the vascular endothelium [24]. Several studies identified CM as a strong predictor of AKI, especially in patients with preexisting renal impairment [25–27]. In our analysis, however, we did not observe an association between CM volume and AKI incidence for either the non-CKD or the CKD cohort. Even for very high CM doses (≥ 300 ml) there was no positive correlation with AKI. A similar finding in a large STEMI cohort of more than 3000 patients was recently reported by Schmucker et al. [28]. The authors speculate that the major cause of AKI in ACS might rather be due to hemodynamic effects based on altered cardiac output during myocardial infarction than to direct renal damage by the CM. Our findings are consistent with this hypothesis. Another study [29] conducted in a much wider setting had similar findings: from a large dataset extracted from the U.S. Nationwide Inpatient Sample comprising nearly 6 million hospitalizations in the year 2009, the risk of contrast agent-associated nephropathy was assessed in the setting of various diseases and pre-existing comorbidities and compared with patients who did not receive contrast media [29]. The rates of AKI were similar (5.5% vs. 5.6%) in the two groups. Interestingly, the subset of patients with ACS who received contrast media had an even lower incidence of AKI compared with those patients who had not received contrast media [29]. Of note, this was a retrospective, non-randomized, yet very large sample-sized study, and possible confounders cannot be ruled out. However, the authors conclude that the so-called “attributable risk”—meaning the risk of AKI that can be attributed to contrast media—is much lower than assumed thus far. Concerning our findings, we speculate one reason for the lack of association between contrast media volume and AKI might be that the physician’s awareness of potential worsening of kidney function is greater for patients who received higher amounts of contrast media or who have preexisting CKD, leading to compensatory treatment strategies (e.g. more extensive fluid administration) compared with patients without CKD or with lower contrast media volume. The latter patients might then be treated in a more cavalier fashion “stepmotherly” leading to a more equal distribution of AKI between these groups.

In conclusion, considering the current data, the role of contrast media in AKI is controversial and can only be adequately addressed by randomized controlled studies. However, such an approach is not feasible and also ethically questionable, as in the case of ACS patients in whom invasive treatment via coronary angiography clearly improves prognosis.

### Limitations

Our analysis has several limitations. Since this is a post-hoc analysis of an existing cohort, follow-up sCR or eGFR measurements were not made within consistent time frames: for every patient, AKI was evaluated by using the first and the last sCR or eGFR value before coronary angiography and before discharge. Thus, the rate of AKI might have been underestimated, given the possibility that renal deterioration in some patients might have peaked beforehand. The alternative *CIN* definition for AKI using a relative rise of baseline sCR of ≥ 25% was not included in the analysis. A further limitation is that the definition of the groups CKD and non-CKD was based solely on the analysis of the first available eGFR value after admission prior to coronary angiography. Such an eGFR value measured during the course of ACS might not always reflect a patient’s “true” kidney function and may possibly have affected the number of patients defined as having CKD used for our analysis. No data on urinary output was available in the evaluated cohort; therefore, criteria of the *AKIN* or *RIFLE* classification regarding urinary output were not investigated.

AKI is a highly complex disease that is influenced by various clinical factors such as hemodynamic changes during the acute or subacute setting of AMI, intravascular volume status, and pre-existing chronic diseases associated with a higher risk of developing AKI (e.g. diabetes). As the focus of this study was on the methodological investigation of four different AKI definitions, the impact of these clinical factors on AKI was not examined.

Finally, it should be mentioned that the study cohort derived from a registry conducted between 2003 and 2005 and thus may appear to be rather “old”; nevertheless, our investigation focuses on patients’ kidney function measured via blood tests that are comparable to assays currently in use. As the CM applied during that period was low-osmolar and is still available on the market, we assume that it was not more detrimental than the CM used currently in the hospital, which is also low-osmolar.

## Conclusion

Our study demonstrates that development of AKI, classified on the basis of four different AKI definitions, is strongly associated with mid-term, all-cause mortality in a real-world ACS cohort. Patients with AKI according to the *CIN* definition had a higher 6-month mortality rate than those with AKI according to the widely used *AKIN* definitions adopted by KDIGO. The amount of CM used showed no association with the development of AKI, irrespective of the AKI definition used or of the presence of CKD.

## Supplementary Information

**Additional file 1: Suppl. Figure 1**. Relationship of volume of contrast media and AKI. Distribution of volume of contrast media according to the development of an AKI based on four different AKI definitions and stratified by CKD status. Abbreviations: CKD = chronic kidney disease; AKI = acute kidney injury. **Suppl. Table 1**. Measured serum creatinine levels and estimated glomerular filtration rate on admission and at discharge stratified by CKD status. **Suppl. Table 2**. Development of AKI according to the four different AKI definitions stratified by CKD status. **Suppl. Table 3**. Discriminatory ability of the estimated glomerular filtration rate at enrolment to identify patients developing AKI according to the four different AKI definitions. **Suppl. Table 4**. Correlations between volume of contrast agent used and development of AKI calculated via Pearson correlation for the different AKI definitions and stratified by CKD.

## Data Availability

The datasets used and/or analyzed during the current study available from the corresponding author on reasonable request.
