# Supplementary material for: Definition of acute kidney injury impacts prevalence and prognosis in ACS patients undergoing coronary angiography
Source: BMC Cardiovasc Disord. 2021 Apr 15;21:183. doi: 10.1186/s12872-021-01985-9 (PMC8051101; doi:10.1186/s12872-021-01985-9)
Supplement: Supplementary file 1 — Additional file 1: Suppl. Figure 1. Relationship of volume of contrast media and AKI. Distribution of volume of contrast media according to the development of an AKI based on four different AKI definitions and stratified by CKD status. Abbreviations: CKD = chronic kidney disease; AKI = acute kidney injury. Suppl. Table 1. Measured serum creatinine levels and estimated glomerular filtration rate on admission and at discharge stratified by CKD status. Suppl. Table 2. Development of AKI according to the four different AKI definitions stratified by CKD status. Suppl. Table 3. Discriminatory ability of the estimated glomerular filtration rate at enrolment to identify patients developing AKI according to the four different AKI definitions. Suppl. Table 4. Correlations between volume of contrast agent used and development of AKI calculated via Pearson correlation for the different AKI definitions and stratified by CKD. [file 12872_2021_1985_MOESM1_ESM.docx]

**Supplemental material**

**Definition of acute kidney injury impacts prevalence and prognosis**

**in ACS patients undergoing coronary angiography**

Maren Weferling^1,2^, MD; Christoph Liebetrau^1,2,3^, MD; Daniel Kraus, MD^4^; Philipp Zierentz^3^;

Beatrice von Jeinsen, MD^1,2^, Oliver Dörr^1,3^, MD; Michael Weber^5^, MD; Holger Nef, MD^1,2,3^; Christian W Hamm^1,2,3^, MD, FESC; Till Keller ^1,2,3,6^, MD, FESC

^1^ Department of Cardiology, Kerckhoff Heart and Thorax Center, Bad Nauheim, Germany

^2^ German Centre for Cardiovascular Research (DZHK), partner site RheinMain, Germany

^3^ University of Giessen, Medical Department I, Cardiology, Giessen, Germany

^4^University of Mainz, Medical Department I, Nephrology, Mainz, Germany

^5^ Hospital Darmstadt-Dieburg, Department of Internal Medicine II, Groß-Umstadt, Germany

^6^Justus-Liebig-Universität Gießen, Campus Kerckhoff, Department of Cardiology, Benekestr. 2-8, 61231 Bad Nauheim, Germany

**Supplement data**

**Suppl. Figure 1: Relationship of volume of contrast media and AKI**

Distribution of volume of contrast media according to the development of an AKI based on four different AKI definitions and stratified by CKD status

Abbreviations: CKD= chronic kidney disease; AKI= acute kidney injury

**Suppl. Table 1**: **Measured serum creatinine levels and estimated glomerular filtration rate on admission and at discharge stratified by CKD status**

**Suppl. Table 2: Development of AKI according to the four different AKI definitions stratified by CKD status**

**Suppl. Table 3**: **Discriminatory ability of the estimated glomerular filtration rate at enrolment to identify patients developing AKI according to the four different AKI definitions**

**Suppl. Table 4: Correlations between volume of contrast agent used and development of AKI calculated via Pearson correlation for the different AKI definitions and stratified by CKD**

**Suppl. Figure 1: Relationship of volume of contrast media and AKI**


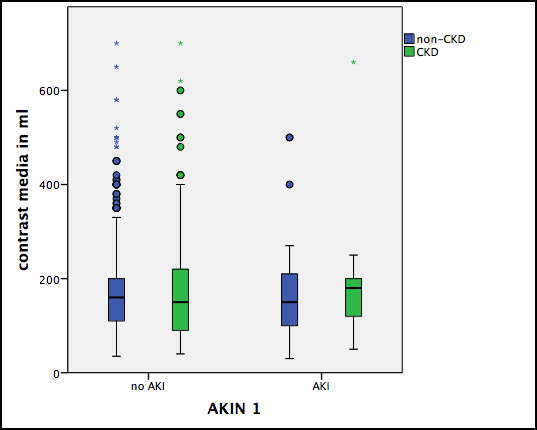

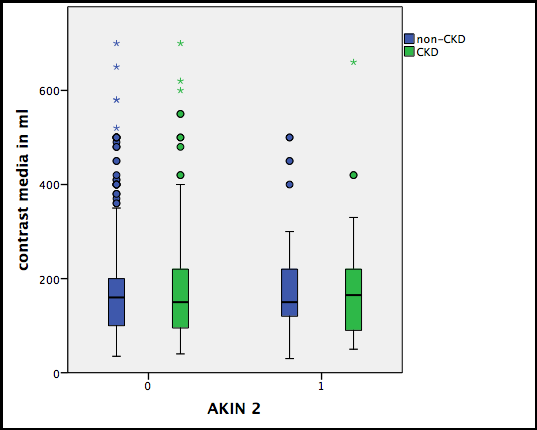


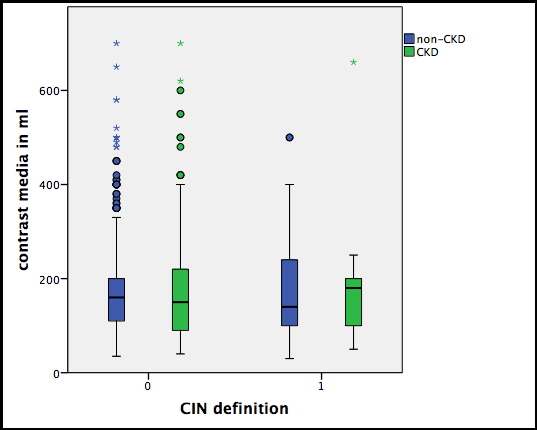

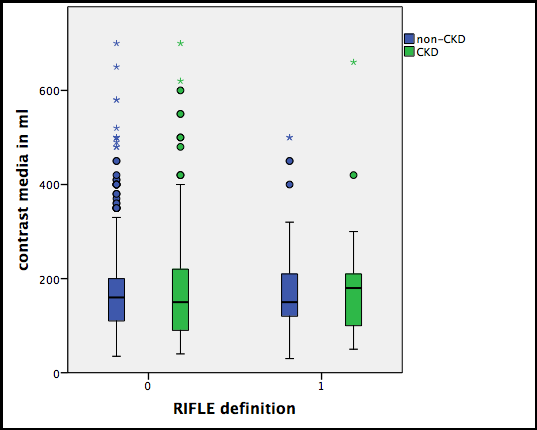


Distribution of volume of contrast media according to the development of an AKI based on four different AKI definitions and stratified by CKD status

Abbreviations: CKD= chronic kidney disease; AKI= acute kidney injury

**Suppl. Table 1**: Measured serum creatinine levels and estimated glomerular filtration rate on admission and at discharge stratified by CKD status

|  | **Entire**  **cohort**  mean (SD) | **non-CKD patients**  mean (SD) | **CKD**  **patients**  mean (SD) | *p*-value |
| --- | --- | --- | --- | --- |
| On admission |  |  |  |  |
| sCR (mg/dl) | 0.98 (0.49) | 0.84 (0.17) | 1.53 (0.84) | *<0.001* |
| eGFR (ml/min/1.73 m^2^) | 80.57 (25.66) | 89.41 (19.98) | 45.47 (11.96) | *<0.001* |
| At discharge |  |  |  |  |
| sCR (mg/dl) | 1.06 (0.53) | 0.93 (0.3) | 1.54 (0.88) | *<0.001* |
| eGFR (ml/min/1.73 m^2^) | 75.13 (24.94) | 81.86 (21.57) | 48.43 (18.81) | *<0.001* |

Abbreviations: eGFR=estimated glomerular filtration rate; sCr= serum creatinine, SD= standard deviation

**Suppl. Table 2:** Development of AKI according to the four different AKI definitions stratified by CKD status

|  | **Entire**  **cohort**  N (%) | **non-CKD patients**  N (%) | **CKD**  **patients**  N (%) | *p*-value |
| --- | --- | --- | --- | --- |
| *AKI development according to* |  |  |  |  |
| AKIN 1 definition | 50 (5.3) | 34 (4.5) | 16 (8.4) | *0.03* |
| AKIN 2 definition | 96 (10.2) | 55 (7.3) | 41 (21.6) | *<0.001* |
| CIN definition | 42 (4.4) | 21 (2.8) | 21 (11.1) | *<0.001* |
| RIFLE definition | 125 (13.2) | 93 (12.3) | 32 (16.8) | 0.101 |

Abbreviations: AKI= acute kidney injury; CKD=chronic kidney disease

**Suppl. Table 3**: Discriminatory ability of the estimated glomerular filtration rate at enrolment to identify patients developing AKI according to the four different AKI definitions

| **Estimated glomerular filtration rate** | **Entire cohort**  **AUC** | **95%CI** | ***p-value*** |
| --- | --- | --- | --- |
| *AKI development according to* |  |  |  |
| AKIN 1 definition | 0.58 | 0.05-0.49 | *0.05* |
| AKIN 2 definition | 0.68 | 0.62-0.74 | *<0.001* |
| CIN definition | 0.75 | 0.67-0.82 | *<0.001* |
| RIFLE definition | 0.5 | 0.44-0.56 | 0.91 |

Abbreviations: AUC= Area under the curve; CI= confidence interval; AKI=acute kidney injury

**Suppl. Table 4:** Correlations between volume of contrast agent used and development of AKI calculated via Pearson correlation for the different AKI definitions and stratified by CKD

|  | **Entire cohort** | | **non-CKD patients** | | **CKD patients** | |
| --- | --- | --- | --- | --- | --- | --- |
|  | Pearson's r | *p*-value | Pearson's r | *p*-value | Pearson's r | *p*-value |
| AKIN 1 definition | 0.011 | 0.74 | 0.006 | 0.87 | 0.015 | 0.83 |
| AKIN 2 definition | 0.016 | 0.63 | 0.024 | 0.52 | -0.017 | 0.82 |
| CIN definition | 0.013 | 0.69 | 0.019 | 0.60 | -0.010 | 0.90 |
| RIFLE definition | -0,004 | 0.90 | -0.002 | 0.96 | -0.017 | 0.82 |

Abbreviations: CKD=chronic kidney disease
